# Adjuvant immunochemotherapy with oral Tegafur/Uracil plus PSK in patients with stage II or III colorectal cancer

**DOI:** 10.1038/sj.bjc.6602100

**Published:** 2004-08-10

**Authors:** C Alliot

**Affiliations:** 1 Hematology/Oncology Division, General Hospital of Annemasse, BP 525, Annemasse, Cedex 74107, France

**Sir**,

In the March 8 issue, Ohwada *et al* (2004) reported a randomised study of patients with stage II or III colorectal cancer, treated with either UFT alone or the combination of UFT and PSK. The benefit obtained by PSK seems impressive, with a decrease by 43.6% in the risk of recurrence and 5-year disease-free survival of 73%, especially as there were only 139 patients in the investigational group and 68 in the control group. Nevertheless, several aspects must be discussed. The first point is the choice of the control arm. The combination of 5-FU and leucovorin currently is the standard adjuvant chemotherapy of colorectal cancer in most countries. There is currently no evidence that UFT, even when modulated by leucovorin, is superior to this regimen. On the contrary, a large phase III trial comparing UFT/leucovorin with 5-FU/LV (Mayo Clinic regimen) in 816 patients with metastatic disease resulted in an increase by 22% in the risk of disease progression in the investigational arm (Douillard *et al*, 2002). Another randomised study showing some benefit in terms of survival for cancer death only used oral 5-FU in the control arm (Ito *et al*, 2004). Of note is the high proportion of patients with rectal cancer, reaching 50% in the control group. Given the impact of the quality of surgery and radiotherapy in this location, a confusing factor might have been introduced especially as preoperative radiotherapy has not been administered. The study design introduces several variables in chemotherapy such as early start, long duration, sequential administration of MMC and UFT, introducing other confusing factors. The early start of chemotherapy might be interesting since surgery provokes the circulation of neoplastic cells (Yamaguchi *et al*, 2000) and angiogenesis and potentially the development of micrometastasis. Intravenous chemotherapy usually starts 4–8 weeks after surgery. Nevertheless, the poor results of the control arm do not support this approach. On a psychological viewpoint, the impact of a very long treatment should not be neglected since it may enforce the idea that cancer can relapse any time. The results in stage III patients in the control arm are poor and question about the efficacy of UFT and MMC. Indirectly, the mediocrity of UFT alone minimises the impact of PSK. The authors largely invoke indirect comparisons with studies published more than 10 years before while many procedures, including surgery, improve over time and promising trials of combination chemotherapy with either oxaliplatin or irinotecan are ongoing. The impact of secondary surgery as well as second-line chemotherapy in well-followed patients may be important in terms of overall survival. The issue of the mechanism of action of PSK, in particular its synergy with a fluoropyrimidine, is intriguing. Since decades, a tremendous number of studies regarding immunotherapy have been reported. Although extracts of microbial agents can logically induce demonstrable production of interleukin-2, interferon, or activated immune cells, evidence of a clinical impact remains extremely limited. Even high-dose interleukin-2 or interferon result only in a modest efficacy in subgroups of patients with cancers partially dependant on immunesurveillance such as advanced renal cancer or melanoma. The efficacy of interferon in the adjuvant therapy of melanoma is highly debated (Sabel and Sondak, 2003). Recent randomised studies have shown that neither interferon (Messing *et al*, 2003) nor interleukin-2 (Clark *et al*, 2003) improve survival in the adjuvant treatment of high-risk renal carcinoma. Consequently, the impact of ‘soft’ immunotherapy is difficult to advocate in colorectal cancer. In addition, several recent randomised studies have called into question the efficacy of levamisole (QUASAR Collaborative Group, 2000; Dencausse *et al*, 2002; Cascinu *et al*, 2003). Probably, an alternative mechanism should be considered. Inhibition of metastases by protection of vascular membrane basement has also been evoked (Kudo *et al*, 2002). In conclusion, although PSK remains a good candidate for convenient adjuvant therapy, this study is not definitively convincing. The challenge remains crucial since oral therapy is particularly adapted to the vast concerned population, particularly in the elderly, given the relatively heavy procedure of combination chemotherapy with FOLFOX or FOLFIRI regimen.
